# Design, Synthesis and Antiviral Activity Studies of Schizonepetin Derivatives

**DOI:** 10.3390/ijms140817193

**Published:** 2013-08-20

**Authors:** Beihua Bao, Zheng Meng, Nianguang Li, Zhengjie Meng, Li Zhang, Yudan Cao, Weifeng Yao, Mingqiu Shan, Anwei Ding

**Affiliations:** 1 Jiangsu Key Laboratory for High Technology of TCM Formulae Research, College of Pharmacy, Nanjing University of Chinese Medicine, Nanjing 210023, China; E-Mails: beihua.bao@gmail.com (B.B.); mengzheng_nj@163.com (Z.M.); linianguang@njutcm.edu.cn (N.L.); raindc@163.com (Y.C.); weifengyao@sohu.com (W.Y.); shanmingqiu@163.com (M.S.); 2 College of Biotechnology and Pharmaceutical Engineering, Nanjing University of Technology, Nanjing 211816, China; E-Mail: rockymeng.jm@gmail.com

**Keywords:** schizonepetin, anti-virus activity, HSV-1, H3N2

## Abstract

A series of schizonepetin derivatives have been designed and synthesized in order to obtain potent antivirus agents. The antiviral activity against HSV-1 and influenza virus H3N2 as well as the cytotoxicity of these derivatives was evaluated by using cytopathic effect (CPE) inhibition assay *in vitro*. Compounds M2, M4, M5 and M34 showed higher inhibitory activity against HSV-1 virus with the TC_50_ values being in micromole. Compounds M28, M33, and M35 showed higher inhibitory activity against influenza virus H3N2 with their TC_50_ values being 96.4, 71.0 and 75.4 μM, respectively. Preliminary biological activity evaluation indicated that the anti-H3N2 and anti-HSV-1 activities improved obviously through the introduction of halogen into the structure of schizonepetin.

## 1. Introduction

Herpes simplex viruses such as HSV-1 and HSV-2 are enveloped DNA viruses, each of which comprises at least 77 genes assigned to four kinetic classes, namely α, β, γ1, and γ2 on the basis of the timing of requirements for their expression. The five α genes, α0, α4, α22, α27, and α47, are expressed firstly in the absence of viral protein synthesis and are responsible for the regulated expression of the other viral genes. The β gene requires functional α gene products for their expression and encode proteins and enzymes that are directly involved in DNA synthesis and nucleotide metabolism. The γ gene forms the last set of viral genes to be expressed, with the γ2 class having viral DNA replication as a strict requirement for their expression [1].

It is common for humans to be infected with HSV viruses that may result in several diseases such as labial herpes, genital herpes, chronic mucocutane-ous ulceration, keratoconjunctivitis, and meningoencephalitis. In certain cases, it may even lead to life-threatening conditions, especially in immunocompromised patients with acquired immunodeficiency syndrome and in organ transplant recipients [2–5].

HSV infections are usually treated with nucleoside analogues such as acyclovir, penciclovir, valacyclovir, famciclovir and adenine arabinoside (ara-A) [6,7]. Acyclovir, valacyclovir and famciclovir can be used to shorten the course and decrease the severity of HSV infections and may suppress the virus itself, thereby preventing future outbreaks especially for the genital herpes [7]. However, the efficacy of these drugs is limited by the recent increase in the resistance of virus and recurrence of latent virus [8–11]. Thus, the development of new anti-HSV agents is highly desirable.

Schizonepetin, a naturally occuring monoterpene featured with a lactone structure, was isolated from the essential oil of *Schizonepeta tenuifolia* [12]. A HPLC method had been developed to determine schizonepetin and its impurities in schizonepetin bulk drug [13]. Our previous study showed that schizonepetin had an obviously positive therapeutic effect on the H_1_N_1_ infected mice, which indicated that schizonepetin may be a good anti-H_1_N_1_ lead compound [14]. It was also proved that schizonepetin could inhibit the increase of abdominal capillary permeability by HAc [15,16]. The acute, subacute and genetic toxicity of schizonepetin has also been assessed [17]. In addition, the pharmacokinetic profiles of schizonepetin and its effects on activity and mRNA expression of cytochrome P450 enzymes in rats has been studied [18,19].

As the antiviral activity of schizonepetin is not strong, it is necessary to modify its structure in order to obtain some schizonepetin derivatives with improved biological activities. Lipid solubility is a key factor to affect the bioavailability of a drug, improve the lipid solubility of a drug is able to enhance the membrane penetration [20]. In the present paper, we have synthesized some ether and ester derivatives of schizonepetin as to increase its lipid solubility and the antiviral activity, the preliminary antiviral activity and cytotoxicity of these synthesized compounds against HSV-1 and influenza virus H3N2 were also tested by using cytopathic effect (CPE) inhibition assay *in vitro*.

## 2. Results and Discussion

### 2.1. Chemistry

**Scheme 1:**
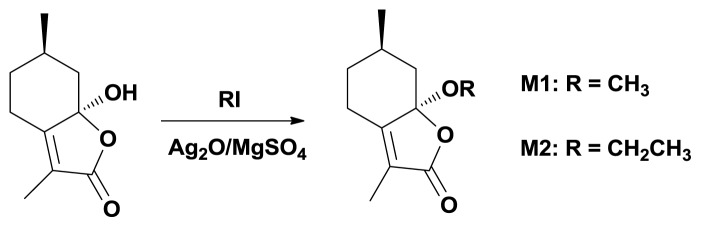
Synthetic route to ether **M1** and **M2**.

The ether derivatives **M1** and **M2** were obtained by the reaction of schizonepetin with alkyl iodides in the presence of silver oxide in CH_3_CN according to the reported procedure as shown in Scheme 1 [21].

**Scheme 2:**
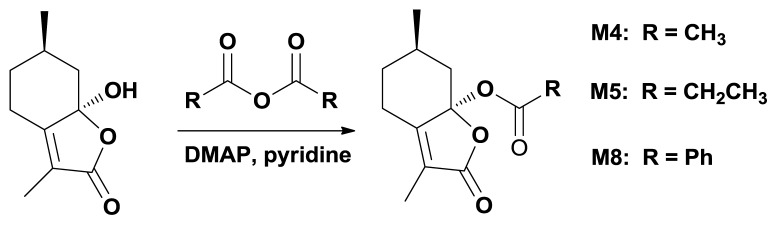
Synthetic route for aliphatic ester **M4**, **M5** and **M8**.

The ester derivatives **M4**, **M5** and **M8** were synthesized by using anhydrides as acylation reagents in pyridine with 4-dimethylaminopyridine (DMAP) as the catalyst as shown in Scheme 2 [22].

**Scheme 3:**
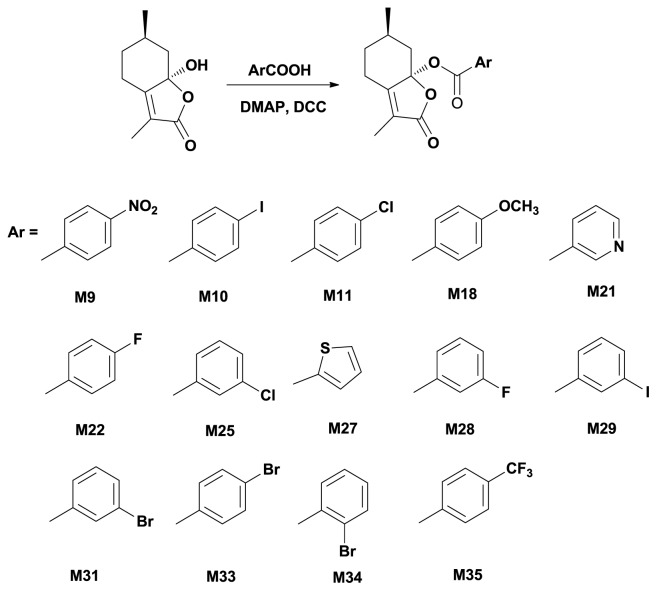
Synthetic route to aromatic ester **M9**–**M35**.

In order to obtain the other ester derivatives **M9**–**M35**, firstly we tried to use anhydrides as acylation reagents, but the reaction failed and no products appeared. Thus anther strategy was used as shown in Scheme 3, under the catalysts of *N*,*N*′-dicyclohexylcarbodiimide (DCC) and DMAP. The reaction proceeded smoothly to give the target compounds **M9**–**M35** in high yields.

### 2.2. Biological Activity

All the prepared compounds were evaluated for their inhibitory effects and cytotoxicity against the HSV-1 virus and influenza virus H3N2 *in vitro* by CPE inhibition assay, with aciclovir and ribavirin used as the reference drugs respectively. The results were summarized in Table 1.

As shown in Table 1, it could be concluded that the schizonepetin had low antiviral activity against HSV-1 and influenza virus H3N2 *in vitro*. However, its derivatives M2, M4, M5, and M34 showed obvious antiviral activity against HSV-1 virus, while aromatic ester derivatives M28, M33, and M35 showed high antiviral activity against influenza virus H3N2.

Structure-activity analysis on these derivatives revealed which substituent was required for the antiviral activity. With IC_50_ value being 100.0 μM and TC_50_ value being 246.7 μM against HSV-1, the ether compound **M2** was more active than compound **M1**, whose TC_50_ value was 366.8 μM. With IC_50_ value was 26.9 μM and TC_50_ value was 66.0 μM against HSV-1, the ester compound **M5** was more active than compound **M4** with its IC_50_ value was 93.8 μM and TC_50_ value was 374.6 μM respectively. These results indicated that activity against HSV-1 might be improved when the carbon chain increased. Among aromatic esters, p-bromophenyl ester compound **M34** showed the excellent antiviral activity against HSV-1 with its IC_50_ value being 17.1 μM. However the o-bromophenyl ester compound **M31** and m-bromophenyl ester compound **M33** had little effect on antiviral activity against HSV-1. The compound **M34** showed the best antiviral activity against HSV-1 (IC50 = 17.1 μM) as shown in Table 1.

From the results of antiviral activity against influenza virus H3N2 shown in Table 1, we found that the m-bromophenyl ester compound **M33** showed excellent antiviral activity against influenza virus H3N2 among aromatic esters with its IC_50_ value being 13.7 μM. However the o-bromophenyl ester compound **M31** and p-bromophenyl ester compound **M34** showed little effect on antiviral activity against influenza virus H3N2. The other m-bromophenyl esters, such as compounds **M10**, **M11** and **M22**, also showed little effect on antiviral activity against influenza virus H3N2. Among o-bromophenyl esters, compound **M28** showed obvious antiviral activity against influenza virus H3N2 with its IC_50_ value being 34.5 μM. The other compounds **M25**, **M29** and **M31** showed little effect on antiviral activity against influenza virus H3N2. The m-bromophenyl esters such as compound **M35** with its IC_50_ value being 29.7 μM showed stronger activity than compound M9. The compound **M33** exhibited the best antiviral activity against influenza virus H3N2 (IC_50_ = 13.7 μM).

Preliminary biological activity evaluation indicated that introduction of halogen into the structure of schizonepetin, the anti-HSV-1 and anti-H3N2 activity clearly improved.

These results strongly suggested that compounds **M33** and **M34** could be considered as promising candidates for the development of new derivatives with anti-HSV-1 and anti-H3N2 activities and for additional studies concerning the antiviral activity of this group of compounds.

## 3. Experimental Section

### 3.1. Chemistry

All reagents were obtained from commercial sources and were used as received. Solvents were dried and purified by using standard techniques. Reactions were monitored by TLC. Melting points were uncorrected. ^1^H NMR spectra were recorded on a Brucker ACF-300 MHz spectrometer in CDCl_3_. Mass spectra were recorded on HPLC-MS (Waters2695-Q-Tof micro mass spectrometer).

### 3.2. General Procedure for the Preparation of **M1** and **M2**

A solution of schizonepetin (29.2 mg, 0.16 mmol), Ag_2_O (185.4 mg, 0.8 mmol) and MgSO_4_ (100 mg) was dissolved in CH_3_CN (5 mL) and the solution was cooled to 0–4 °C. Alkyl iodide 0.1 mL was added drop wise to the former solution. The reaction mixture was stirred at room temperature. The solution was filtered and the solvent was removed *in vacuo* to afford crude product, which was purified by Prep TLC (Pet/EtOAc = 6:1) to give compounds **M1** and **M2**.

#### 3.2.1. (6*R*,7a*R*)-7a-Methoxy-3,6-Dimethyl-5,6,7,7a-Tetrahydrobenzofuran-2(4H)-One (**M1**)

Yield 58%; H^1^-NMR (300MHz, CDCl_3_): δ 0.95 (d, 3H, *J* = 6.6 Hz, 6′-CH_3_), 1.05 (dd, 1H, *J* = 4.2, 13.2 Hz, 5a-H ), 1.25 (m, 1H, 4a-H), 1.80 (s, 3H, 3′-H), 1.94 (m, 1H, 5b-H), 2.01 (m, 1H, 6-H), 2.13(m, 1H, 4b-H), 2.41 (m, 1H, 7a-H), 2.70 (m, 1H, 7b-H), 3.12 (s, 3H, -OCH_3_); ESI-MS: *m*/*z* 197 [M + H]^+^.

#### 3.2.2. (6*R*,7a*R*)-7a-Ethoxy-3,6-Dimethyl-5,6,7,7a-Tetrahydrobenzofuran-2(4H)-One (**M2**)

Yield 58%; H^1^-NMR (300MHz, CDCl_3_): δ 0.96 (d, 3H, *J* = 6.6 Hz, 6′-CH_3_), 1.07 (dd, 1H, *J* = 4.2, 13.2 Hz, 5a-H ), 1.18 (t, 3H, *J* = 7.2, -O-C-CH_3_), 1.26 (m, 1H, 4a-H), 1.84 (s, 3H, 3′-H), 1.95 (m, 1H, 5b-H), 1.98 (m, 1H, 6-H), 2.20 (m, 1H, 4b-H), 2.41 (m, 1H, 7a-H), 2.70 (m, 1H, 7b-H), 3.21–3.39 (m, 2H, -OCH_2_-); ESI-MS: *m*/*z* 211 [M + H]^+^.

### 3.3. General Procedure for the Preparation of **M4**, **M5** and **M8**

A solution of Schizonepetin (29.2 mg, 0.16 mmol) in 5 mL pyridine was added 5 mL anhydride and DMAP (0.16 mmol, 1.0 eq). The reaction mixture was stirred at room temperature for 6 h, then poured into 50 mL of ethyl acetate, and the mixture was washed with 150 mL water 3 times. The organic extracts were concentrated on a rotary evaporator afford crude product, which was purified by Prep TLC (Pet/EtOAc = 7:1) to give title compounds.

#### 3.3.1. (6*R*,7a*R*)-3,6-Dimethyl-2-oxo-2,4,5,6,7,7a-Hexahydrobenzofuran-7a-Yl Acetate (**M4**)

Yield 87%; H^1^-NMR (300MHz, CDCl_3_): δ 0.98 (d, 3H, *J* = 6.6 Hz, 6′-CH_3_), 1.05 (dd, 1H, *J* = 4.2, 13.2 Hz, 5a-H ), 1.26 (m, 1H, 4a-H), 1.85 (s, 3H, 3′-H), 1.95 (m, 1H, 5b-H), 1.99 (m, 1H, 6-H), 2.05 (s, 3H, -CO-CH_3_), 2.17(m, 1H, 4b-H), 2.71 (m, 2H, 7-H); ESI-MS: *m*/*z* 225 [M + H]^+^.

#### 3.3.2. (6*R*,7a*R*)-3,6-Dimethyl-2-oxo-2,4,5,6,7,7a-Hexahydrobenzofuran-7a-yl Propionate (**M5**)

Yield 84%; H^1^-NMR (300MHz, CDCl_3_): δ 0.98 (d, 3H, *J* = 6.6 Hz, 6′-CH_3_), 1.05 (dd, 1H, *J* = 4.2, 13.2 Hz, 5a-H ), 1.1 (t, 3H, *J* = 7.5 Hz, -CO-C-CH_3_), 1.26 (m, 1H, 4a-H), 1.87 (s, 3H, 3′-H), 1.96 (m, 1H, 5b-H), 1.99 (m, 1H, 6-H), 2.15(m, 1H, 4b-H), 2.33 (m, 2H, -CO-CH2-R), 2.71 (m, 2H, 7-H); ESI-MS: *m*/*z* 239 [M + H]^+^.

#### 3.3.3. (6*R*,7a*S*)-3,6-Dimethyl-2-Oxo-2,4,5,6,7,7a-Hexahydrobenzofuran-7a-yl Benzoate (**M8**)

Yield 93%; H1-NMR (300MHz, CDCl_3_): δ 1.02 (d, 3H, *J* = 6.6 Hz, 6′-CH_3_), 1.05 (dd, 1H, *J* = 4.2, 13.2 Hz, 5a-H ), 1.28 (m, 1H, 4a-H), 1.91 (s, 3H, 3′-H), 2.05 (m, 2H, 5b-H, 6-H), 2.24 (m, 1H, 4b-H), 2.84 (m, 2H, 7-H), 7.42–7.47 (m, 2H, Ar-H), 7.59 (m, 1H, Ar-H), 7.98–8.01 (m, 2H, Ar-H); ESI-MS: *m*/*z* 287 [M + H]^+^.

### 3.4. General Procedure for the Preparation of **M9**–**M35**

A solution of schizonepetin (29.2 mg, 0.16 mmol), aromatic carbonyl acid (0.32 mmol, 2.0 eq) and DMAP (0.16 mmol, 1.0 eq) was dissolved in CH_2_Cl_2_ (5 mL), DCC (0.32 mmol, 2.0 eq) was added drop wise to the former solution. The reaction mixture was stirred at room temperature. The organic extracts were concentrated on a rotary evaporator affording a crude product, which was purified by Prep TLC (Pet/EtOAc = 5:1) to give title compounds.

#### 3.4.1. (6*R*,7a*S*)-3,6-Dimethyl-2-Oxo-2,4,5,6,7,7a-Hexahydrobenzofuran-7a-yl 4-Nitrobenzoate (**M9**)

Yield 67%; H^1^-NMR (300MHz, CDCl_3_): δ 1.05 (d, 3H, *J* = 6.6 Hz, 6′-CH_3_), 1.14 (dd, 1H, *J* = 4.2, 13.2 Hz, 5a-H ), 1.26 (m, 1H, 4a-H), 1.92 (s, 3H, 3′-H), 1.95–2.06 (m, 2H, 5b-H, 6-H), 2.22(m, 1H, 4b-H), 2.85 (m, 2H, 7-H), 8.15 (dt, 2H, *J* = 5.1, 9.0 Hz, Ar-H), 8.29 (dt, 2H, *J* = 5.1, 9.0 Hz, Ar-H); ESI-MS: *m*/*z* 332 [M + H]^+^.

#### 3.4.2. (6*R*,7a*S*)-3,6-Dimethyl-2-Oxo-2,4,5,6,7,7a-Hexahydrobenzofuran-7a-yl 4-Iodobenzoate (**M10**)

Yield 73%; H^1^-NMR (300MHz, CDCl_3_): δ 1.03 (d, 3H, *J* = 6.6 Hz, 6′-CH_3_), 1.09 (dd, 1H, *J* = 4.2, 13.2 Hz, 5a-H), 1.26 (m, 1H, 4a-H), 1.84 (s, 3H, 3′-H), 1.93–2.02 (m, 2H, 5b-H, 6-H), 2.21(m, 1H, 4b-H), 2.78–2.86 (m, 2H, 7-H), 7.67 (dt, 2H, *J* = 1.2, 2.4 Hz, Ar-H), 7.81 (dt, 2H, *J* = 1.2, 2.4 Hz, Ar-H); ESI-MS: *m*/*z* 413 [M + H]^+^.

#### 3.4.3. (6*R*,7a*S*)-3,6-Dimethyl-2-Oxo-2,4,5,6,7,7a-Hexahydrobenzofuran-7a-yl 4-Chlorobenzoate (**M11**)

Yield 89%; H^1^-NMR (300MHz, CDCl_3_): δ 1.05 (d, 3H, *J* = 6.6 Hz, 6′-CH_3_), 1.14 (dd, 1H, *J* = 4.2, 13.2 Hz, 5a-H ), 1.26 (m, 1H, 4a-H), 1.92 (s, 3H, 3′-H), 1.95–2.05 (m, 2H, 5b-H, 6-H), 2.23(m, 1H, 4b-H), 2.79–2.90 (m, 2H, 7-H), 7.43 (dt, 2H, *J* = 2.1, 9.0 Hz, Ar-H), 7.93 (dt, 2H, *J* = 2.1, 9.0 Hz, Ar-H); ESI-MS: *m*/*z* 321.5 [M + H]^+^.

#### 3.4.4. (6*R*,7a*S*)-3,6-Dimethyl-2-Oxo-2,4,5,6,7,7a-Hexahydrobenzofuran-7a-yl 4-Methoxybenzoate (**M18**)

Yield 86%; H^1^-NMR (300MHz, CDCl_3_): δ 1.02 (d, 3H, *J* = 6.6 Hz, 6′-CH_3_), 1.09 (dd, 1H, *J* = 4.2, 13.2 Hz, 5a-H ), 1.27 (m, 1H, 4a-H), 1.90 (s, 3H, 3′-H), 1.95–2.01 (m, 2H, 5b-H, 6-H), 2.24 (m, 1H, 4b-H), 2.77–2.86 (m, 2H, 7-H), 3.86 (s, 3H, -OCH_3_), 6.91 (dt, 2H, *J* = 1.5, 2.7 Hz, Ar-H), 7.94 (dt, 2H, *J* = 1.5, 2.7 Hz, Ar-H); ESI-MS: *m*/*z* 317 [M + H]^+^.

#### 3.4.5. (6*R*,7a*S*)-3,6-Dimethyl-2-Oxo-2,4,5,6,7,7a-Hexahydrobenzofuran-7a-yl Nicotinate (**M21**)

Yield 89%; H^1^-NMR (300MHz, CDCl_3_): δ 1.04 (d, 3H, *J* = 6.6 Hz, 6′-CH_3_), 1.08 (dd, 1H, *J* = 3.0, 9.6 Hz, 5a-H ), 1.26 (m, 1H, 4a-H), 1.92 (s, 3H, 3′-H), 1.97–2.04 (m, 2H, 5b-H, 6-H), 2.24(m, 1H, 4b-H), 2.80–2.89 (m, 2H, 7-H), 7.40–9.19 (m, 4H, -C_6_NH_4_); ESI-MS: *m*/*z* 288 [M + H]^+^.

#### 3.4.6. (6*R*,7a*S*)-3,6-Dimethyl-2-Oxo-2,4,5,6,7,7a-Hexahydrobenzofuran-7a-yl 4-Fluorobenzoate (**M22**)

Yield 82%; H^1^-NMR (300MHz, CDCl_3_): δ 1.05 (d, 3H, *J* = 6.6 Hz, 6′-CH_3_), 1.16 (dd, 1H, *J* = 4.2, 13.2 Hz, 5a-H ), 1.26 (m, 1H, 4a-H), 1.92 (s, 3H, 3′-H), 1.96–2.03 (m, 2H, 5b-H, 6-H), 2.24(m, 1H, 4b-H), 2.78–2.88 (m, 2H, 7-H), 7.10–8.04 (m, 4H, Ar-H); ESI-MS: *m*/*z* 305 [M + H]^+^.

#### 3.4.7. (6*R*,7a*S*)-3,6-Dimethyl-2-Oxo-2,4,5,6,7,7a-Hexahydrobenzofuran-7a-yl 3-Chlorobenzoate (**M25**)

Yield 88%; H^1^-NMR (300MHz, CDCl_3_): δ 1.05 (d, 3H, *J* = 6.6 Hz, 6′-CH_3_), 1.11 (dd, 1H, *J* = 4.2, 13.2 Hz, 5a-H ), 1.26 (m, 1H, 4a-H), 1.91 (s, 3H, 3′-H), 1.94–2.03 (m, 2H, 5b-H, 6-H), 2.23(m, 1H, 4b-H), 2.78–2.88 (m, 2H, 7-H), 7.39 (t, 1H, *J* = 4.8 Hz, Ar-H), 7.56 (m, 1H, Ar-H), 7.87 (m, 1H, Ar-H), 7.94 (t, 1H, *J* = 0.9 Hz, Ar-H); ESI-MS: *m*/*z* 321.5 [M + H]^+^.

#### 3.4.8. (6*R*,7a*S*)-3,6-Dimethyl-2-Oxo-2,4,5,6,7,7a-Hexahydrobenzofuran-7a-yl Thiophene-2-Carboxylate (**M27**)

Yield 90%; H^1^-NMR (300MHz, CDCl_3_): δ 1.02 (d, 3H, *J* = 6.6 Hz, 6′-CH_3_), 1.08 (dd, 1H, *J* = 4.2, 13.2 Hz, 5a-H ), 1.26 (m, 1H, 4a-H), 1.89 (s, 3H, 3′-H), 1.95–2.04 (m, 2H, 5b-H, 6-H), 2.25(m, 1H, 4b-H), 2.77–2.84 (m, 2H, 7-H), 7.10–7.79 (m, 3H, -C_4_H_3_S); ESI-MS: *m*/*z* 293 [M + H]^+^.

#### 3.4.9. (6*R*,7a*S*)-3,6-Dimethyl-2-Oxo-2,4,5,6,7,7a-Hexahydrobenzofuran-7a-yl 3-Fluorobenzoate (**M28**)

Yield 84%; H^1^-NMR (300MHz, CDCl_3_): δ 1.04 (d, 3H, *J* = 6.6 Hz, 6′-CH_3_), 1.07 (dd, 1H, *J* = 4.2, 13.2 Hz, 5a-H ), 1.29 (m, 1H, 4a-H), 1.91 (s, 3H, 3′-H), 1.94–2.03 (m, 2H, 5b-H, 6-H), 2.23(m, 1H, 4b-H), 2.78–2.87 (m, 2H, 7-H), 7.28–7.79 (m, 4H, Ar-H); ESI-MS: *m*/*z* 305 [M + H]^+^.

#### 3.4.10. (6*R*,7a*S*)-3,6-Dimethyl-2-oxo-2,4,5,6,7,7a-Hexahydrobenzofuran-7a-yl 3-Iodobenzoate (**M29**)

Yield 73%; H^1^-NMR (300MHz, CDCl_3_): δ 1.04 (d, 3H, *J* = 6.6 Hz, 6′-CH_3_), 1.12 (dd, 1H, *J* = 4.2, 13.2 Hz, 5a-H ), 1.28 (m, 1H, 4a-H), 1.90 (s, 3H, 3′-H), 1.94–2.01 (m, 2H, 5b-H, 6-H), 2.23(m, 1H, 4b-H), 2.78–2.88 (m, 2H, 7-H), 7.19 (t, 1H, *J* = 4.8 Hz, Ar-H), 7.90–7.96 (m, 2H, Ar-H), 8.29 (t, 1H, *J* = 0.9 Hz, Ar-H); ESI-MS: *m*/*z* 413 [M + H]^+^.

#### 3.4.11. (6*R*,7a*S*)-3,6-Dimethyl-2-Oxo-2,4,5,6,7,7a-Hexahydrobenzofuran-7a-yl 3-Bromobenzoate (**M31**)

Yield 75%;H^1^-NMR (300MHz, CDCl_3_): δ 1.04 (d, 3H, *J* = 6.6 Hz, 6′-CH_3_), 1.11 (dd, 1H, *J* = 4.2, 13.2 Hz, 5a-H ), 1.28 (m, 1H, 4a-H), 1.91 (s, 3H, 3′-H), 1.93–2.03 (m, 2H, 5b-H, 6-H), 2.23(m, 1H, 4b-H), 2.78–2.88 (m, 2H, 7-H), 7.33 (t, 1H, *J* = 4.8 Hz, Ar-H), 7.71 (m, 1H, Ar-H), 7.92 (dt, 1H, *J* = 0.9, 4.8 Hz, Ar-H), 8.09 (t, 1H, *J* = 0.9 Hz, Ar-H); ESI-MS: *m*/*z* 366 [M + H]^+^.

#### 3.4.12. (6*R*,7a*S*)-3,6-Dimethyl-2-Oxo-2,4,5,6,7,7a-Hexahydrobenzofuran-7a-yl 4-Bromobenzoate (**M33**)

Yield 78%; H^1^-NMR (300MHz, CDCl_3_): δ 1.04 (d, 3H, *J* = 6.6 Hz, 6′-CH_3_), 1.11 (dd, 1H, *J* = 4.2, 13.2 Hz, 5a-H ), 1.28 (m, 1H, 4a-H), 1.92 (s, 3H, 3′-H), 1.94–2.04 (m, 2H, 5b-H, 6-H), 2.23(m, 1H, 4b-H), 2.78–2.87 (m, 2H, 7-H), 7.58 (dt, 2H, *J* = 1.2, 5.4 Hz), 7.84 (dt, 2H, *J* = 1.2, 5.4 Hz); ESI-MS: *m*/*z* 366 [M + H]^+^.

#### 3.4.13. (6*R*,7a*S*)-3,6-Dimethyl-2-Oxo-2,4,5,6,7,7a-Hexahydrobenzofuran-7a-yl 2-Bromobenzoate (**M34**)

Yield 86%; H^1^-NMR (300MHz, CDCl_3_): δ 1.01 (d, 3H, *J* = 6.6 Hz, 6′-CH_3_), 1.11 (dd, 1H, *J* = 4.2, 13.2 Hz, 5a-H ), 1.26 (m, 1H, 4a-H), 1.90 (s, 3H, 3′-H), 1.99–2.10 (m, 2H, 5b-H, 6-H), 2.37(m, 1H, 4b-H), 2.79–2.86 (m, 2H, 7-H), 7.32–7.39 (m, 2H, Ar-H), 7.65 (dd, 1H, *J* = 0.9, 4.5 Hz, Ar-H), 7.75 (dd, 1H, *J* = 1.2, 4.5 Hz, Ar-H); ESI-MS: *m*/*z* 366 [M + H]^+^.

#### 3.4.14. (6*R*,7a*S*)-3,6-Dimethyl-2-Oxo-2,4,5,6,7,7a-Hexahydrobenzofuran-7a-yl 4-(Trifluoromethyl) Benzoate (**M35**)

Yield 83%; H1-NMR (300MHz, CDCl_3_): δ 1.07 (d, 3H, *J* = 6.6 Hz, 6′-CH3), 1.13 (dd, 1H, *J* = 4.2, 13.2 Hz, 5a-H ), 1.32 (m, 1H, 4a-H), 1.93 (s, 3H, 3′-H), 1.96–2.07 (m, 2H, 5b-H, 6-H), 2.27(m, 1H, 4b-H), 2.80–2.94 (m, 2H, 7-H), 7.62 (t, 1H, *J* = 7.8 Hz, Ar-H), 7.86 (d, 1H, *J* = 7.8 Hz, Ar-H), 8.19 (d, 1H, *J* = 7.8 Hz, Ar-H), 8.24 (s, 1H, Ar-H); ESI-MS: *m*/*z* 355 [M + H]^+^.

### 3.5. Biological Activity

#### 3.5.1. Cytotoxicity Studies

The cytotoxicity was evaluated by cytopathic effect (CPE) inhibition assay. Vero cells were seeded into 96-well plates. After cells had been incubated for 24 h at 37 °C in 5% CO_2_, they were exposed to drugs with two-fold serial dilutions. Untreated Vero cells were used as controls. All cultures were incubated again in 5% CO_2_ at 37 °C for 72 h. The cells were examined microscopically for the cytotoxicity effects. Then TC_50_ were calculated by the Reed-Muench method.

#### 3.5.2. Anti HSV-1 Studies

The antiviral activities were determined by CPE inhibition assay. Vero cells were seeded into 96-well plates and incubated for 24 h at 37 °C in 5% CO_2_. After infected with 30 TCID_50_ virus solutions, cultures were incubated for another 2 h at 37 °C in 5% CO_2_, and then the supernatants of HSV-1 were discarded. Subsequently, 100 μl various concentrations of compounds were added to quadruplicate culture wells. Cells infected with HSV-1 incubated only with medium and cells infected with HSV-1 incubated with various concentrations of acyclovir were used as controls. All cultures were incubated again in 5% CO_2_ at 37 °C for 72 h. The cells were examined microscopically for CPE and 50% inhibitory concentration (IC_50_) and therapeutic index (TI) were calculated.

#### 3.5.3. Anti-H3N2 Studies

The procedures for Anti-H3N2 studies and cytotoxicity against MDCK cells *in vitro* by CPE inhibition assay were similar to Anti HSV-1 studies and cytotoxicity against Vero cells.

## 4. Conclusions

In conclusion, we designed and synthesized two series of schizonepetin derivatives in order to find some compounds with higher antivirus activity than the parent compound schizonepetin. Two compounds, **M33** and **M34**, emerged as potential antiviral agents. Preliminary biological activity evaluation of these derivatives with electron-withdrawing substituents on schizonepetin indicated that introduction of halogen into the structure of schizonepetin, anti-H3N2 and anti-HSV-1 activity clearly improved. The effects of electron-donating substituents and further structural modifications of schizonepetin are in progress and will be reported in due course.

## Figures and Tables

**Table 1:** *In vitro* antiviral activity against HSV-1and H3N2 virus of schizonepetin derivatives.

Entry	Compounds	Antiviral activity against HSV-1 virus			Antiviral activity against H3N2 virus		
	
		TC_50_(μM)	IC_50_(μM)	TI	TC_50_(μM)	IC_50_(μM)	TI
1	**M1**	366.8	-		298.5	-	-
2	**M2**	246.7	100.0	2.5	139.5	-	-
3	**M4**	374.6	93.8	4.0	319.2	-	-
4	**M5**	66.0	26.9	2.5	39.1	-	-
5	**M8**	24.8	-	-	54.2	-	-
6	**M9**	216.0	-	-	216.0	-	-
7	**M10**	44.2	-	-	41.3	-	-
8	**M11**	47.1	-	-	47.1	-	-
9	**M21**	439.0	-	-	180.5	-	-
10	**M22**	87.8	-	-	60.5	-	-
11	**M25**	11.5	-	-	80.8	-	-
12	**M27**	100.3	-	-	177.4	-	-
13	**M28**	170.4	-	-	96.4	34.5	2.8
14	**M29**	45.4	-	-	71.1	-	-
15	**M31**	80.3	-	-	80.3	-	-
16	**M33**	80.3	-	-	71.0	13.7	5.2
17	**M34**	80.3	17.1	4.7	71.0	-	-
18	**M35**	82.8	-	-	75.4	29.7	2.5
19	Schizonepetin	834.2	-	-	829.4	-	-
20	Aciclovir	>666.0	4.9	>136.4	ND	ND	ND
21	Ribavirin	ND	ND	ND	ND	1023.7	-

-: no inhibitory effects on virus at the concentration of TC_50_; TI = TC_50_/IC_50_; ND: not determined.
